# Mathematical modeling clarifies the paracrine roles of insulin and glucagon on the glucose-stimulated hormonal secretion of pancreatic alpha- and beta-cells

**DOI:** 10.3389/fendo.2023.1212749

**Published:** 2023-08-14

**Authors:** Aedan Brown, Emmanuel S. Tzanakakis

**Affiliations:** ^1^ Department of Chemical and Biological Engineering, Tufts University, Medford, MA, United States; ^2^ Genetics, Molecular and Cellular Biology, Tufts University, Boston, MA, United States; ^3^ Pharmacology and Drug Development, Tufts University, Boston, MA, United States; ^4^ Clinical and Translational Science Institute, Tufts University, Boston, MA, United States

**Keywords:** pancreas, diabetes, insulin, glucagon, beta-cells, alpha-cells, modeling

## Abstract

**Introduction:**

Blood sugar homeostasis relies largely on the action of pancreatic islet hormones, particularly insulin and glucagon. In a prototypical fashion, glucagon is released upon hypoglycemia to elevate glucose by acting on the liver while elevated glucose induces the secretion of insulin which leads to sugar uptake by peripheral tissues. This simplified view of glucagon and insulin does not consider the paracrine roles of the two hormones modulating the response to glucose of α- and β-cells. In particular, glucose-stimulated glucagon secretion by isolated α-cells exhibits a Hill-function pattern, while experiments with intact pancreatic islets suggest a ‘U’-shaped response.

**Methods:**

To this end, a framework was developed based on first principles and coupled to experimental studies capturing the glucose-induced response of pancreatic α- and β-cells influenced by the two hormones. The model predicts both the transient and steady-state profiles of secreted insulin and glucagon, including the typical biphasic response of normal β-cells to hyperglycemia.

**Results and discussion:**

The results underscore insulin activity as a differentiating factor of the glucagon secretion from whole islets *vs*. isolated α-cells, and highlight the importance of experimental conditions in interpreting the behavior of islet cells *in vitro*. The model also reproduces the hyperglucagonemia, which is experienced by diabetes patients, and it is linked to a failure of insulin to inhibit α-cell activity. The framework described here is amenable to the inclusion of additional islet cell types and extrapancreatic tissue cells simulating multi-organ systems. The study expands our understanding of the interplay of insulin and glucagon for pancreas function in normal and pathological conditions.

## 1 Introduction

The pancreatic islets of Langerhans are central to the regulation of blood glucose through the release of hormones, mainly insulin and glucagon (1). Insulin-releasing β-cells are the most common species in the islets, while the glucagon-secreting α-cells make up most of the remaining cells (2). While blood glucose acts as the primary signal for these cells, the secreted moieties also influence intra-islet hormonal responses creating a multi-layered signaling landscape. Elevated glucose stimulates β-cells, while it appears to inhibit α-cells (3). Completing a feedback loop, insulin causes the uptake of glucose by cells in the muscle, liver and fat whereas glucagon stimulates gluconeogenesis releasing glucose from the liver. As a second layer of paracrine interactions, insulin and glucagon influence the function of α- and β-cells, respectively (4, 5). It is suggested that insulin inhibits α-cells’ ability to release glucagon, while glucagon activates insulin secretion by β-cells (2, 3, 6–8). These interactions present challenges in understanding the relative importance of α- and β-cells in blood sugar control under normal and disease conditions.

Much of the previous work on pancreatic islets has focused on β-cells given their central role in diabetes. In type 1 diabetes (T1D), β-cells, which are 55% of the human islet cell population (2), are ablated due to autoimmunity, whereas type 2 diabetes (T2D) is linked to damage of β-cells and reduction in their mass due to insulin resistance exhibited by peripheral tissues (9). A step increase in glucose concentration *in vivo* or *in vitro* causes a biphasic response by β-cells with an initial surge of insulin release followed by a steady-state plateau. In T2D however, β-cells lose the initial peak *in vivo* and exhibit a more muted response *in vitro* (9, 10). Hence, being a hallmark of normalcy, the biphasic secretion pattern has been observed experimentally and has guided the development of relevant computational constructs (11–13). As these computational efforts have elucidated our understanding of β-cells, further experimental work is focused on clarifying the functional regulation of α-cells in the islets.

The crosstalk between β-cells and α-cells, which comprise 40% (2) of human islet cells, has potential implications on the hormonal response to glucose. Many computational models assume that glucose exclusively acts as an inhibitor of glucagon secretion by α-cells, as seen in pharmacology (14, 15). This assumption is supported by experiments examining ion channel activity (16, 17), intracellular Ca^2+^ levels (18), and cAMP levels (19) in α-cells within islets. Yet, a U-shape response has been reported for intact islets: at low and high glucose levels, glucagon secretion is relatively high but not at midrange (18, 20), suggesting a more nuanced interpretation may be necessary. Glucose activates glucagon secretion in isolated rat (21) and mouse (22) α-cells, as well as seemingly having no impact on clonal mice α-cells (23), which might be opposite of the prevailing view of glucagon as a key counterregulatory hormone that prevents hypoglycemia by increasing hepatic glucose output. One possible explanation for this ambiguity is that many experiments supporting glucose-suppression of glucagon secretion were collected in a batch setting: the islets were incubated for a fixed period with constant glucose level (16–18). Because intact islets were used, glucagon and insulin, as well as other key islet species (e.g., somatostatin, GABA) were continuously secreted, and their concentrations varied throughout the experiment. Thus, the crosstalk among islet cells may confound the true effect of blood sugar on islet output. The lack of consensus around the exact action of α-cells, a key player in glucose regulation, warrants a more thorough exploration of the role of these cells within the pancreatic islet.

Like the biphasic insulin response, mathematical models have also been used to clarify the role of α-cells, both independent from (24) and within the islets (25–29). These models have highlighted the importance of paracrine interactions for proper islet functions, as well as glucose’s central role. However, connecting many of these models with commonly measured experimental data (i.e., glucose, glucagon, and insulin concentrations) is difficult due to either not directly considering one of these variables, or modeling abstract “activity” levels of the individual cells. One model of the α-cell (24) has recapitulated the U-shape of glucagon secretion, suggesting glucose could act exclusively as an inhibitor. However, intraislet paracrine interactions were not considered, which are absent in isolated dispersed α-cells.

Here, we set out to elucidate the interactions among α- and β-cells and their effects on hormone secretion upon exposure to glucose. To this end, a mathematical framework was developed based on first principles and in conjunction with data from published experiments. Using the perspective that glucose stimulates glucagon secretion, the model is aligned with results obtained *in vitro*, where islet paracrine interactions can be isolated. Moreover, known qualitative interactions are captured between glucagon and β-cells and insulin and α-cells. Among the outputs is the biphasic response of healthy β-cells. Importantly, our findings highlight insulin action as a source of the discrepancy between glucagon secretion from islets and isolated α-cells. This further supports the notion that the hyperglucagonemia seen in T1D and T2D is linked to a failure of insulin (due to β-cell ablation) to inhibit α-cell activity. Overall, our study shines light on the physiological role of α-cells in normal glucose homeostasis or from the perspective of aberrant pancreatic function.

## 2 Materials and methods

### 2.1 Model development

**Figure 1:**
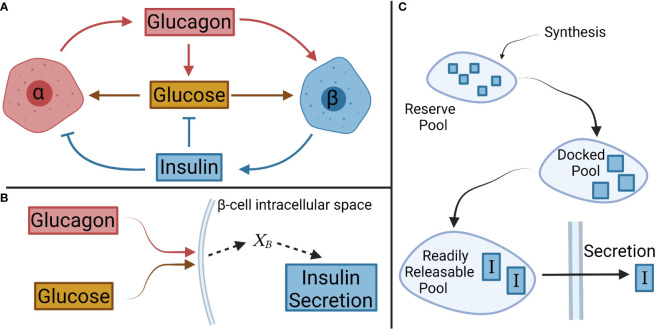
Schematic of interactions between glucose, and α- and β-cells, and their respective hormones. **(A)** Overall interactions between glucagon, insulin, and glucose considered in the model development. **(B)** In β-cells, glucagon and glucose combine to form the net signal, X_B_, which drives insulin secretion. A similar logic is applied to α-cells with insulin and glucose creating a net signal X_A_. **(C)** A pool model describes the secretion kinetics.

Our effort centered on the network comprising insulin, glucagon, and glucose among α- and β-cells which make almost 90% of the islet cells (**Figure 1A**). The model development was divided in two parts: First, the steady-state behavior of the system was captured. Second, a transient, kinetic model was constructed describing how the system approaches steady state. Finally, mass balances were performed on key species to relate secretion and bulk solution concentration.

#### 2.1.1 Steady-state model

The steady-state portion of the model was developed involving the interactions of glucose with α-cells and β-cells, and their steady-state hormonal secretion. A net signal was assumed to determine the secretion, as shown schematically for β-cells in **Figure 1B**. This signal will be denoted $X_{B}$ for β-cells and $X_{A}$ for α-cells. Equations 1 and 2 describe the mass secretion rate of insulin, $R_{I}$ , and glucagon, $R_{G}$ , as functions of $X_{B}$ and $X_{A}$ , respectively.


(1) $$R_{I}\left(X_{B}\right)=\frac{m_{I}X_{B}^{n_{I}}}{X_{B}^{n_{I}}+h_{I}^{n_{I}}}$$

(2) $$R_{G}\left(X_{A}\right)=\frac{m_{G}X_{A}^{n_{G}}}{X_{A}^{n_{G}}+h_{G}^{n_{G}}}$$

$R_{G}$ was cast as a Hill function based on the glucagon results from isolated α-cells. A Hill function was also considered for $R_{I}$ , as this trend is observed for insulin secretion in both batch and perifusion experiments (3, 30). Of note, both glucagon and glucose contribute positively to insulin secretion leading to the same effect of the potential crosstalk. Thus, the trend captured in experiments *in vitro* is likely accurate. Furthermore, Hill type relations have been employed by other groups to describe steady-state insulin secretion (11, 12). Equations 1 and 2 relate the steady-state secretion to the net signals, $X_{B}$ and $X_{A}$ .

Next, the net signals as functions of their appropriate secretagogues were determined. Glucagon and glucose levels dictate insulin secretion, but because their values can vary over orders of magnitude — around 5 mM for glucose and between 5-25 pM for glucagon — a normalized signal was used (31–33). For a generic species *i* at steady state, with [*i*] representing its current concentration and ${\left[i\right]}_{ba}$ representing its basal concentration, the signal is $X_{i}=\left[i\right]/{\left[i\right]}_{ba}$ ensures that one signal does not completely dominate the secretion output due to its absolute value. Equation 3 describes $X_{B}$ as a function of glucose and glucagon signals, $X_{gB}$ and $X_{G}$ , respectively.


(3) $$X_{B}=X_{gB}+\left(\frac{m_{GB}X_{G}^{n_{GB}}}{X_{G}^{n_{GB}}+h_{GB}^{n_{GB}}}\right)\left(\frac{X_{gB}^{n_{gB}}}{X_{gB}^{n_{gB}}+h_{gB}^{n_{gB}}}\right)+X_{B0}$$

Essentially, $X_{B}$ is proportional to $X_{gB}$ , and $X_{G}$ acts to adjust the signal intensity, in a saturating manner that can be turned on or off depending on the glucose signal intensity. Additionally, there is a background signal, $X_{B0}$ , to compensate for secretion that is seen when no glucose is present (18). The $X_{B}$ signal serves as input for insulin secretion (Eq. 1).

A similar equation was developed for $X_{A}$ combining the effects of glucose which induces glucagon secretion and insulin that dampens it:


(4) $$X_{A}=X_{gA}-\frac{\left(m_{g}X_{gA}+X_{A0}\right)X_{I}^{n_{IA}}}{X_{I}^{n_{IA}}+h_{IA}^{n_{IA}}}+X_{A0}$$

As in Equation 3, $X_{A}$ is proportional to the glucose signal $X_{gA}$ . Because $X_{gA}$ and $X_{gB}$ represent the intracellular glucose signal in α- and β-cells, respectively, these values could be different depending on the rate of signal transduction, even for the same extracellular glucose. The insulin signal intensity $X_{I}$ reduces $X_{A}$ in a saturating manner, and $X_{A0}$ represents a basal background signal. The $m_{g}$ term limits how much insulin can remove the glucose signal. The existence of such a limitation is suggested by the U-shape of glucagon secretion. The Appendix contains more information on the derivation of these equations. With Equations 1–4, the steady-state model is fully developed; given glucose, glucagon, and insulin concentrations, and the various model parameters, the glucagon and insulin secretion rates at steady-state can be calculated.

#### 2.1.2 Kinetic model

Next, the transient, kinetic model was developed containing two sections: one for simulation of the secretion of insulin and glucagon and another representing the transduction of the signals defined above.

##### 2.1.2.1 Dynamic secretion model

A compartmental model was considered for the secretion of insulin based on different pools reflecting the progression of the hormone from the cell interior to the cytoplasmic membrane. We contemplated three key pools (**Figure 1C**): a reserve pool, a docked pool, and a readily releasable pool (34). The reserve pool is supplied by insulin synthesis, and the hormone transitions to the docked pool (34). Insulin generation was not simulated (11, 12) given the large size of the reserve pool containing ample insulin for release in response to a normal increase in extracellular glucose (9). As such, the transition rate from the reserve pool to the docked pool was set to the previously defined $R_{I}$ . This also ensures that the secretion rate determined by $R_{I}$ is achieved at steady state.

The rates of change in the mass of insulin in the docked (*I_1_*) and readily releasable pools (*I_2_*), were modeled as


(5) $$\frac{dI_{1}}{dt}=R_{I}\left(X_{B}\right)-k_{1}\left(X_{B}\right)I_{1}$$

(6) $$\frac{dI_{2}}{dt}=k_{1}\left(X_{B}\right)I_{1}-k_{2}\left(X_{B}\right)I_{2}$$

The rate coefficients *k_1_* and *k_2_* for these transitions were initially described as generic functions of $X_{B}$ , based on how glucose and glucagon signals modulate insulin secretion (2). These functions were determined by examining insulin secretion kinetics in perifusion experiments. As glucose concentrations increase, the kinetic response becomes saturated: eventually, the kinetics do not vary much with glucose (35). This trend suggested that rate coefficients could be modeled as Hill functions:


(7) $$\frac{dI_{1}}{dt}=R_{I}\left(X_{B}\right)-\frac{m_{I1}X_{B}^{n_{I1}}}{h_{I1}^{n_{I1}}+X_{B}^{n_{I1}}}I_{1}$$

(8) $$\frac{dI_{2}}{dt}=\frac{m_{I1}X_{B}^{n_{I1}}}{h_{I1}^{n_{I1}}+X_{B}^{n_{I1}}}I_{1}-\frac{m_{I2}X_{B}^{n_{I2}}}{h_{I2}^{n_{I2}}+X_{B}^{n_{I2}}}I_{2}$$

Glucagon secretion was examined next. Unlike the insulin release kinetics, much less is known about the temporal evolution of α-cell response, which may be transduced in a similar manner to that of β-cells (21) and use similar exocytotic mechanisms (36). Others have reported that glucagon exhibits a biphasic pattern when sugar levels are lowered (37). Thus, the change in glucagon mass within α-cells was modeled similarly to the three-pool model of insulin in β-cells, i.e.,


(9) $$\frac{dG_{1}}{dt}=R_{G}\left(X_{A}\right)-\frac{m_{G1}X_{A}^{n_{G1}}}{h_{G1}^{n_{G1}}+X_{A}^{n_{G1}}}G_{1}$$

(10) $$\frac{dG_{2}}{dt}=\frac{m_{G1}X_{A}^{n_{G1}}}{h_{G1}^{n_{G1}}+X_{A}^{n_{G1}}}G_{1}-\frac{m_{G2}X_{A}^{n_{G2}}}{h_{G2}^{n_{G2}}+X_{A}^{n_{G2}}}G_{2}$$

with *G_1_* and *G_2_* being the glucagon mass in the second and third pools, respectively. The pool model captures the qualitative trends observed experimentally for glucagon secretion.

##### 2.1.2.2 Signal transduction model

While the characteristics of insulin release have been captured in various models for β-cells, the signal transduction that initiates the secretion remains underappreciated. For instance, time delay functionals were employed for the rate of glucose-induced mobilization of insulin granules (12). However, the use of time delay alone ignores the potential influence that more nuanced kinetics of the signal transduction, such as transient signal buildup, could have on insulin secretion.

In the stimulus-secretion coupling network, glucose enters the cell through glucose transporters, and undergoes normal metabolism (2) increasing the ATP to ADP ratio (9). At high levels of ATP, the K_ATP_ channels close, limiting K^+^ efflux (9) and inducing the influx of Ca^2+^ (2) eventually triggering insulin secretion (2). Using a mass-action kinetic model of this network and assuming the transfer of Ca^2+^ is a rate-limiting step, the following equation can be derived for the signal propagation of glucose in β-cells, as shown in the Appendix, where [*g*] is extracellular glucose concentration and $k_{gB}$ is a rate constant for glucose signal transduction:


(11) $$\frac{dX_{gB}}{dt}=k_{gB}\left(\frac{\left[g\right]}{{\left[g\right]}_{ba}}-X_{gB}\right)$$

Conversely, Equations 12, 13, and 14 describe the transduction of glucagon in β-cells ( $X_{G}$ ), glucose in α-cells ( $X_{gA}$ ), and insulin in α-cells ( $X_{I}$ ), respectively. Here, $k_{G}$ , $k_{gA}$ , $k_{I}$ are transduction rate constants, whereas [*G*] and [*I*] represent the concentration of glucagon and insulin, respectively.


(12) $$\frac{dX_{G}}{dt}=k_{G}\left(\frac{\left[G\right]}{{\left[G\right]}_{ba}}-X_{G}\right)$$

(13) $$\frac{dX_{gA}}{dt}=k_{gA}\left(\frac{\left[g\right]}{{\left[g\right]}_{ba}}-X_{gA}\right)$$

(14) $$\frac{dX_{I}}{dt}=k_{I}\left(\frac{\left[I\right]}{{\left[I\right]}_{ba}}-X_{I}\right)$$

While previous work has used a first-order model (11) or time delay (12) to describe a lag in the start of insulin secretion, in our analysis this delay is directly linked to the signal propagation within the cell. Incorporation of signal transduction is essential to understand how insulin and glucagon influence each other as paracrine signals.

### 2.2 Parameter estimation

Experimental data from literature were used to estimate all parameters in the model. This was done by minimizing the sum of squared errors (SSE) of the model prediction compared to the experimentally obtained points. Due to glucagon and insulin concentrations varying over orders of magnitude, the experimental data were used to normalize the residual. This minimization process was performed using either a trust-region-reflective algorithm or an interior-point constrained minimization algorithm (38, 39). The latter algorithm was implemented when there were a high number of parameters to determine, so a scatter search algorithm generated multiple initial guesses to search for a global minimum.

Basal levels of glucose, insulin, and glucagon were obtained from literature (30, 33, 40). All insulin-related kinetic parameters and steady-state parameters (both interaction and secretion) were estimated from literature perifusion data by SSE minimization as described above. As will be discussed further, glucagon-related kinetic parameters were considered as equal to the corresponding insulin parameters. This assumption initially resulted in glucagon secretion trajectories qualitatively different from those observed in Zhu et al. (8), so $k_{gA}$ and $k_{I}$ were scaled to match the qualitative responses.

## 3 Results

### 3.1 Model parameterization

**Figure 2:**
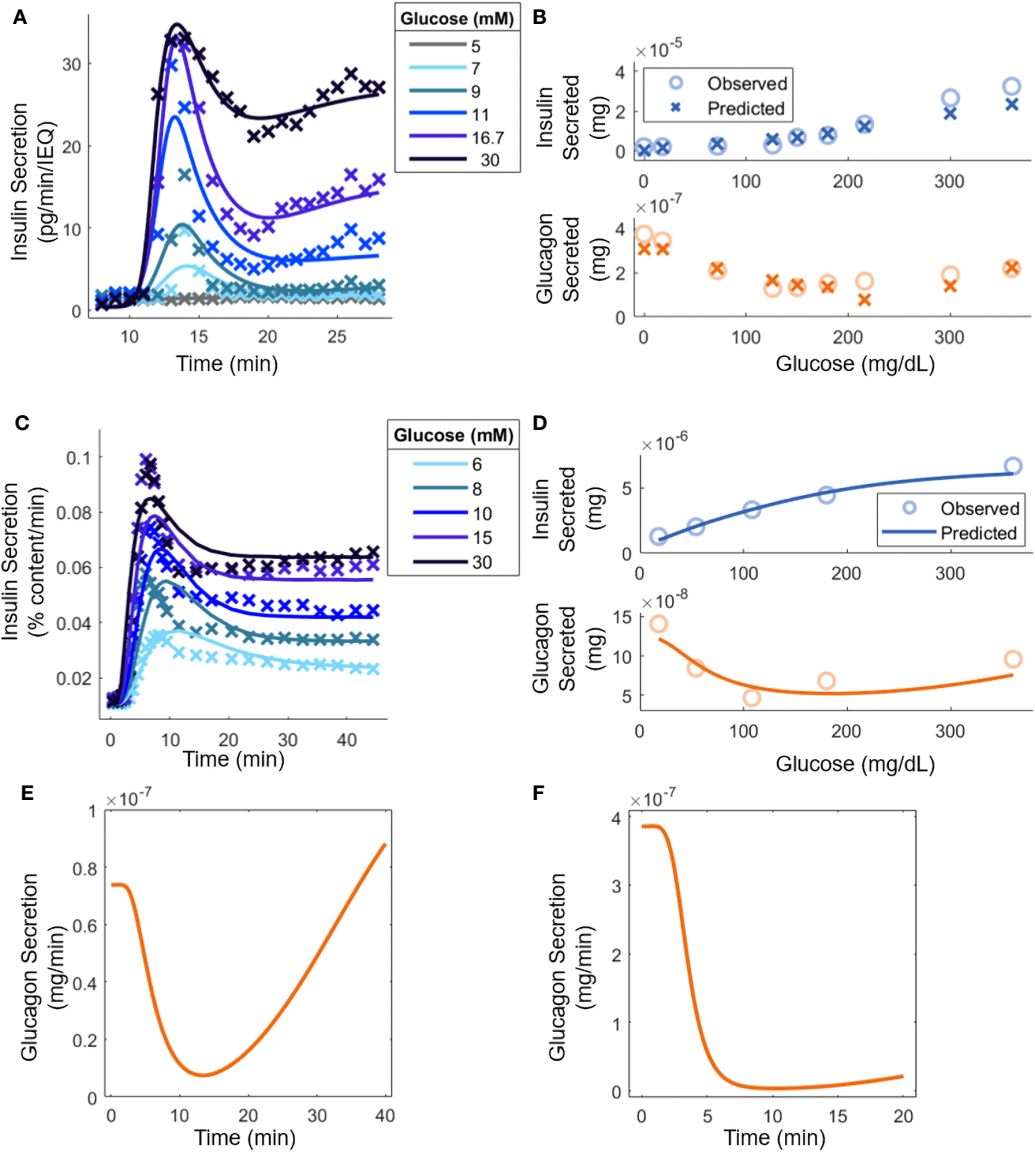
Model parameter evaluation based on experimental data from: **(A)** Mouse islets in a perifusion setting [(30); 70 IEQ]. Model predictions are shown (lines) along with relevant experimental data (points). At 8 minutes, glucose was increased from 3 mM to the indicated level. **(B)** Mouse islets in batch mode (18); 8-12 islets). **(C)** Human islets [(35); 15 islets] under perifusion subjected to an increase in glucose from 3 mM to the stated concentration at t = 0. **(D)** Human islets in batch mode [(20); 10-20 islets]. **(E)** Qualitative comparison to experimental glucagon secretion for a step increase from 3 mM to 16.7 mM glucose with human islets under perifusion [(8), 500 islets]. **(F)** Qualitative comparison to experimental glucagon secretion by human islets for a step increase from 1.8 mM to 7.2 mM in perifusion, which, as explained in the text, is equivalent to the experiment in (8) where 50 mouse islets were exposed to a step increase from 3 mM to 12 mM glucose (50 mouse islets *in vitro*; 5,000 human islets *in silico*).

The constructed model entails 32 parameters, and their values were determined based on published experimental data (8, 18, 20, 30, 35). First, kinetic parameters (Equations 7, 8, and 11) were calculated from studies using mouse islets under perifusion (dynamic) conditions. Then, the steady-state parameters (Equations 1-4) were estimated from data in batch (static) experiments. Similarly, kinetic and steady-state parameters for human islets were computed from measurements obtained in dynamic and static experiments, respectively (**Figure 2**). The values of specific interaction parameters estimated for mouse islet cells were used for the corresponding parameters of human islets and an interior point constrained minimization algorithm was applied to minimize the error between the model predictions and the data. **Supplemental Figure 1** summarizes this workflow. It should be noted that the available reports for parameter estimation differed in the mode of hormonal response interrogation (static *vs*. dynamic) and the number of islets or islet equivalents (IEQ) used (**Figure 2**).

**Figure 2A** shows the model response superimposed to perifusion data from mouse islets. Insulin secretion can heavily inhibit glucagon secretion in perifusion experiments, so it was assumed that $R_{G}\approx 0$ (8), resulting in $X_{gB}$ being the only signal that contributes to insulin secretion ( $X_{B}\approx X_{g\text{B}}+X_{B0}$ because $X_{G}\approx 0$ ). The steady-state parameters in Equation 1, as well as $X_{B0}$ , were determined separately by fitting the steady-state secretion values (**Supplemental Figure 2A**). The SSE was minimized for each trajectory using the interior-point constrained minimization algorithm. Additionally, because the ultimate goal of using the mouse islet data was to determine the interaction parameters in Equations 3 and 4, a set of parameters (Equations 7, 8, and 11) was calculated for each glucose concentration. This allowed tracking the experimentally determined response at each sugar level and ensuring that the steady-state parameters are accurately ascertained. The steady-state parameters (Equations 1-4) were then determined (**Figure 2B**) assuming that insulin and glucagon kinetics (Equations 9, 10) had equal parameters, i.e., $m_{I1}=m_{G1}$ , $h_{I1}=h_{G1}$ , etc and the remaining signal transduction rate constants (Equations 12-14) were equal to $k_{gB}$ . For a given glucose concentration, the kinetic parameters from the closest glucose level in **Figure 2A** were used (the 5 mM result was not used, as the change in insulin secretion was negligible). The steady-state parameters based on the data in **Supplemental Figure 2A** were computed again to account for differences in the experimental methods, such as the media used. During this step, constraints were applied based on available reported results. For example, analysis of the results in Zhu et al. (8) (**Supplemental Table 1**), indicated that $h_{GB}$ could be as large as 1000, so $h_{GB}$ was constrained in the range [500, 1000]. Similarly, $h_{IA}$ values were limited to [1, 100]. At least in the case of $h_{GB}$ , experimental work further confirms this, as nM concentrations of glucagon are needed to stimulate insulin secretion (7, 41), compared to the pM basal concentrations. The normalized SSE was minimized using the interior-point constrained minimization algorithm. The parameter estimation led to a reasonable agreement between the model and the experimental secretion levels for insulin and glucagon. Importantly, the U-shape trend in glucagon response with increasing glucose concentration is recapitulated.

**Figure 2C** shows the model prediction along with the underlying human islet data. As with the mouse islets, it was assumed that $R_{G}\approx 0$ . Again, the steady-state parameters in Equation 1 and $X_{B0}$ were extracted separately from the kinetic data (**Supplemental Figure 2B**). Because of challenges associated with the 5 mM glucose step in **Figure 2A**, it was decided that a weighted SSE should be used, with higher glucose concentration values given larger weights. The weights were 1/15, 2/15, 3/15, 4/15, and 5/15 for 6 mM to 30 mM of glucose. The errors in each trajectory were multiplied by this value before calculating the SSE and minimizing with the interior-point constrained minimization algorithm. Importantly, a single set of parameters described the kinetics in the entire glucose range with the steady-state secretion values predicted on the correct time scale.

Moreover, the steady-state parameters were estimated with batch data (**Figure 2D**). Again, the parameters for glucagon kinetics were assumed to be equal to those used for insulin kinetics. All the interaction parameters from mouse islet data were held constant except for $n_{IA}$ and $h_{IA}$ . Again, $h_{IA}$ was constrained between [8, 100], based on analysis of previous data (8). With parameter adjustment, the glucagon and insulin secretion at any experimentally tested glucose level could be calculated (**Figure 2B**). As before, there is a good quantitative agreement for insulin secretion, and the qualitative U-shape is captured for glucagon production stimulated by glucose. In **Figure 2E**, human islets exposed to a step increase in glucose concentration caused glucagon secretion to drop initially but it eventually increased back to its original level (8). In **Figure 2F**, a setting with mouse islets was surveyed. Because the model was adapted to the hormonal response of human islets, the step change in glucose was normalized by the basal glucose level in mice, i.e., the glucose change from 3 mM to 12 mM was re-scaled to a 1.8 mM to 7.2 mM transition, given the difference in the glycemic set point in mice and humans (42). Additionally, the concentration of insulin present was equated by carrying out the calculations with 5000 islets. In Zhu et al. (8), this scenario led to consistently lower glucagon secretion levels over the period examined, likely a result of increased insulin secretion. The parameter values are shown in **Table 1**.

**Table 1:** Table of parameters used in this model.

Kinetic Parameters				Steady-State Parameters			
				Interaction		Secretion	
k_gB_ (1/min)	0.554	h_I2_	0.968	m_GB_	1.11	m_I_ (pg/min/15 islets)	103
k_G_ (1/min)	0.554	n_I2_	6.68	h_GB_	502	h_I_	3.97
k_gA_ (1/min) *	0.022	m_G1_ (1/min) *	0.336	n_GB_	0.63	n_I_	4.84
k_I_ (1/min) *	2.77	h_G1_ *	3.75	h_gB_	1.07	m_G_ (pg/min/15 islets)	2.24
m_I1_ (1/min)	0.336	n_G1_ *	9.97	n_gB_	0.35	h_G_	1.06
h_I1_	3.75	m_G2_ (1/min) *	0.360	h_IA_	10.0	n_G_	3.5
n_I1_	9.97	h_G2_ *	0.968	n_IA_	1.17	X_B0_	2.60
m_I2_ (1/min)	0.360	n_G2_ *	6.68	m_g_	0.60	X_A0_	4.40

Parameters are dimensionless as they relate to normalized signals unless otherwise noted. Kinetic parameters refer to those included in Equations 7 – 14. Interaction parameters primarily refer to those in Equation 3 and 4, except for X_B0_ and X_A0_, which are included as secretion parameters along with those in Equations 1 and 2. Interaction and secretion parameters are all parameters related to the steady-state secretion. * Parameters estimated from non-curve fit steps such as equating values of glucagon secretion-relevant parameters to those of corresponding parameters for insulin production.

Sensitivity analysis was performed to understand which parameters most influence insulin and glucagon secretion in a perifusion setting. Each parameter was multiplied by a factor ranging from 0.66 to 1.5, one at a time, and the total insulin and glucagon secretion of 15 islets (same as in **Figures 2C, D**) was calculated in response to an increase from 1 mM to 15 mM of glucose under perifusion (**Supplemental Figure 3**). This range was selected, as $m_{g}$ is 0.60 and cannot exceed 1, so 1.5 was chosen as an upper limit, and the reciprocal was taken to achieve a lower bound. Insulin secretion was most greatly affected by $m_{I}$ , $h_{I}$ , $h_{I1}$ , and $n_{I1}$ . The response sensitivity to $m_{I}$ and $h_{I}$ is expected as these directly influence insulin secretion (Eq. 1). The parameters $h_{I1}$ and $n_{I1}$ are involved in the transition of insulin from the first to the second pool (Eq. 7) and control where and how the Hill function describing the rate coefficients increases most. The total secretion likely depends on this regime, because if the rate coefficients are already saturated, there will be minimal change in total secretion. However, if the kinetics are minimally saturated (higher $h_{I1}$ and $n_{I1}$ ), stimulation by glucose will greatly increase the rate coefficients (Eq. 7), magnifying secretion. This notion may not apply to the second transition, as it will be rate-limited by the first transition, potentially explaining the low sensitivity to $h_{I2}$ and $n_{I2}$ . Glucagon secretion was sensitive to $m_{g}$ and $h_{G1}$ . The same reasoning used to explain the sensitivity of the related parameters for insulin secretion likely carries over to the role of these parameters in glucagon secretion. Unexpectedly, the release of insulin and glucagon increased as $X_{B0}$ and $X_{A0}$ , respectively, decreased. A possible explanation is that the system starts at steady state, and with fast kinetics (greater $X_{B0}$ and $X_{A0}$ ), so there is less insulin and glucagon in the pools initially resulting in lower release overall.

### 3.2 Islet number and batch *vs*. perifusion mode on islet hormonal profile

**Figure 3:**
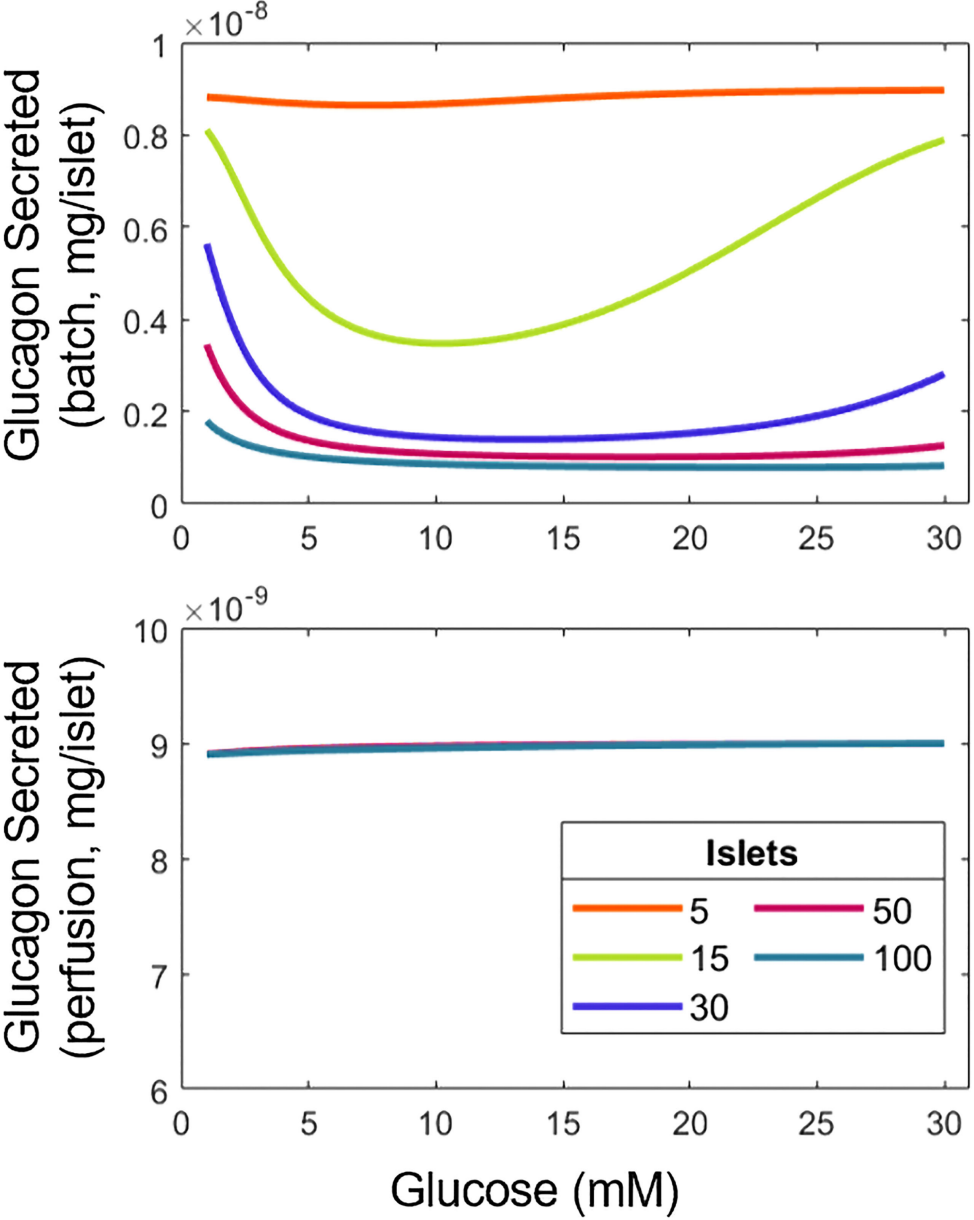
Variation of experimental conditions influences the interpretation of results for the relationship between glucagon and glucose. Total glucagon secretion in batch (top) and perifusion (bottom) modes with various numbers of islets in response to a step increase in glucose from 1 mM to the concentration indicated.

The model parameters were estimated based on data from studies differing in the number of islets used per experiment, and the implementation of static (batch) or dynamic (perifusion) conditions. Generally, *in vitro* experiments utilize 10-15 islets, but in some studies as many as 500 islets were used (8, 18). Hence, the impact of these different experimental factors on the response of α- and β-cells was explored. To allow for comparisons among these conditions, the total insulin or glucagon secreted for an hour-long experiment in a 1 mL chamber was calculated. For dynamic experiments, a perifusion rate of 1 mL/min was used. Contrary to the insulin response (**Supplemental Figure 4**), the release of glucagon was affected significantly by changing the number of islets or conducting perifusion *vs*. batch studies (**Figure 3**).

In batch mode, the U-shape response for glucagon with respect to glucose is observed. However, as the number of islets goes up, glucagon release decreases at low and high glucose concentrations, most likely due to the higher overall amount of insulin produced by the larger number of islets, suppressing the U-type response. Indeed, more insulin means that $X_{I}$ increases, reducing $X_{A}$ and $R_{G}$ (Equations 2, 4). In contrast, the amount of glucagon discharged during perifusion remains flat across the tested range of glucose, likely due to the clearance of insulin abolishing its inhibiting effect on glucagon secretion. **Supplemental Figure 5** helps confirm this, as the U-shape reappears at a lower perifusion rate. Taken together, our findings illustrate the importance of assay conditions, namely batch *vs*. dynamic mode and the number of islets used on the hormonal response of α- and β-cells.

### 3.3 Model application to whole pancreas secretion of insulin and glucagon

**Figure 4:**
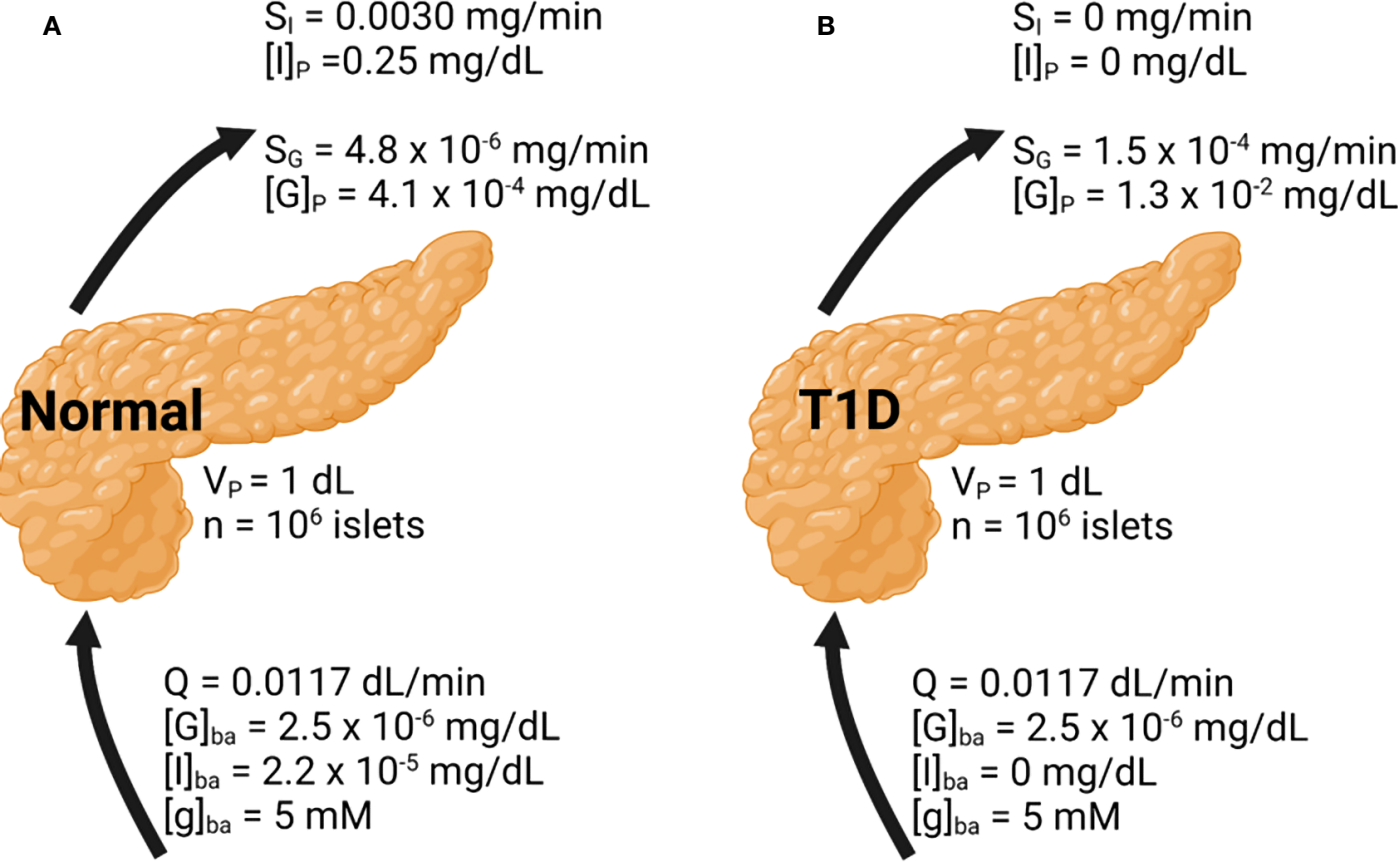
Whole pancreas simulation of the glucose, insulin, and glucagon profiles. Basal conditions are indicated. Results are shown under **(A)** normal and **(B)** T1D conditions.

While this framework was developed using *in vitro* results, we attempted to simulate with it (perifusion mode) an *in vivo* setting. The pancreas volume is approximately 1 dL containing around 10^6^ islets (43, 44). Based on a weight of 90 g, and a blood flow rate of 1.3 mL/min/100 g tissue, the perifusion rate was calculated to be 1.17 mL/min (45, 46). Basal concentrations of glucose, insulin, and glucagon were assumed to be flowing in (**Figure 4**).

In **Figure 4A**, the basal insulin and glucagon secretion rates were predicted to be 3.0 × 10^-3^ mg/min and 4.8 × 10^-6^ mg/min, respectively. The insulin secretion rate agrees well with a value close to 10^-3^ mg/min observed both *in vitro* and *in vivo* (30, 33). The insulin concentration within the pancreas is around 0.25 mg/dL, which greatly diminishes glucagon secretion accounting for its low secretion rate. The concentration of glucagon within the pancreas is low as well, at 4.1 × 10^-4^ mg/dL. While there is less data to confirm the glucagon secretion rate, the agreement in the prediction of insulin secretion supports the validity of our approach. **Figure 4B** illustrates the model results in T1D. To approximate this setting, $m_{I}$ in Equation 1 and the basal insulin level were set to 0. Without any source of insulin, the α-cell side of the model will progress as if β-cells were not present. The calculated glucagon secretion rate is 1.5 × 10^-4^ mg/min and the calculated pancreatic glucagon concentration is 30-fold higher than in the normal pancreas, in line with the hyperglucagonemia observed in patients with diabetes (47).

### 3.4 Interplay of glucagon and insulin on α- and β-cell hormone secretion

**Figure 5:**
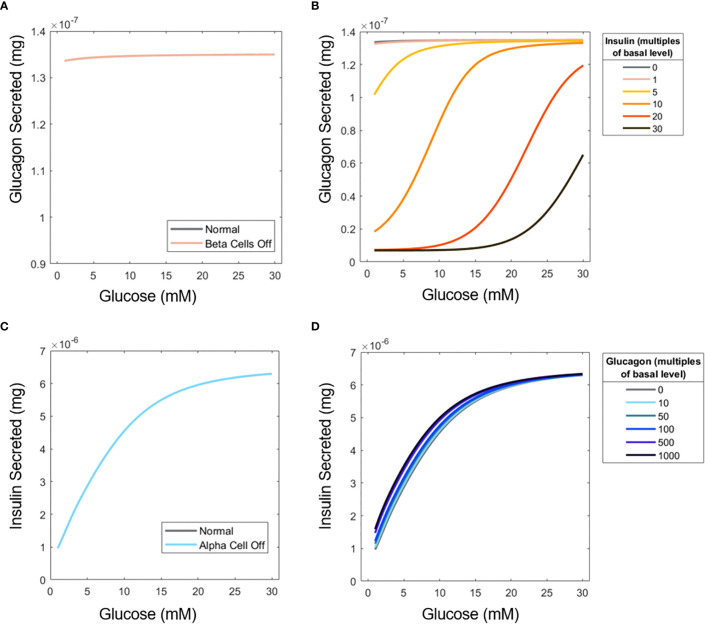
Influence of insulin on glucagon secretion and vice versa. **(A)** ‘Elimination’ of β-cells to examine the effect of insulin on α-cell glucagon secretion. **(B)** Glucagon profiles of islets under different concentrations of perifused exogenous insulin. **(C)** ‘Elimination’ of α-cells to assess the influence of glucagon on β-cell secretion. **(D)** Insulin profiles for islets perifused with exogenous glucagon. Simulations were performed with 15 islets.

Next, we investigated the interplay of α-cells and β-cells in the context of their hormonal production. To this end, α-cells or β-cells were eliminated (by setting $m_{G}=0$ or $m_{I}=0$ ) to see how insulin or glucagon secretion would change, respectively, in pure populations of each cell type (**Figure 5A**). As in **Figure 3**, the U-shape response does not manifest because of the low number of islets in perifusion mode.

Furthermore, a scenario was considered in which extra insulin and glucagon are supplied into the perifusion chamber with both cell types active. While the U-shape is not recovered when insulin is added to the inlet flow, likely because of the uniform effect of additional insulin (**Figure 5B**), the inhibitory effects of insulin on glucagon secretion are apparent. Contrary to the significant impact of insulin on glucagon secretion, glucagon is shown to have a minimal impact on insulin secretion. **Figure 5C** shows that when α-cells are absent, insulin secretion barely changes, again likely due to the small number of islet cells examined, and the fact that $m_{GB}$ is relatively low at 1.11. Adding extra glucagon to the system (**Figure 5D**) has a more pronounced effect at low glucose. Glucose likely becomes the primary inducer of insulin secretion at higher concentrations, so the effects of glucagon are less pronounced.

## 4 Discussion

While various models have been reported to describe the secretion of insulin by islet cells (13), the release of glucagon and its role as an intraislet paracrine signal remain underappreciated. Given the lack of consensus regarding the influence of glucose on glucagon secretion (18, 21), we built a model to elucidate the interplay of glucagon and insulin on the glucose-stimulated response of α- and β-cells. Using this framework, we were able to recapitulate the glucagon secretion influenced by glucose conforming to the experimentally documented U-shape. Parameters such as the number of islet cells and static or dynamic mode of assaying hormone secretion are principal, and their effects will be explored in future studies in greater detail. Additionally, this work showcases quantitatively the hyperglucagonemia seen in T1D as a consequence of the elimination of β-cells and thus of insulin’s inhibitory effect on glucagon secretion.

In previous studies, the translocation of insulin within β-cells was simulated utilizing intracellular hormone ‘pools’ with different states (primed *vs*. unprimed). For example, a three-pool model was constructed featuring both forward and backward transitions and assuming a heterogeneous population of β-cells (11). A five-compartment system was also proposed with two exclusively forward paths to the readily-releasable pool of insulin (12). Here, a three-pool model with only forward transitions was implemented. Besides its simplicity, this scheme captures qualitatively the biphasic pattern of glucose-stimulated insulin secretion (GSIS). Moreover, insulin release peaks at the same time at all concentrations of glucose tested (**Figures 2A, B**). This suggests that certain rate constants in the pool model may be invariable with extracellular glucose concentrations. Interestingly, the same multi-pool concept was applied to the secretion of glucagon here, as done elsewhere (27, 28), and the resulting framework reproduced the hormone production by human α-cells with high fidelity. To achieve these results, α-cell kinetic parameters were equated to the respective β-cell parameters. This assumption is likely valid, as the kinetics of insulin and glucagon release play out over similar timescales (8), and the underlying physiological secretion processes are similar (36). Additionally, the sensitivity analysis (**Supplemental Figure 3**) confirms that the model is relatively insensitive to most of the kinetic parameters, further validating this approach. Nonetheless, the modeling effort presented here will benefit from additional experimental studies designed to extract specifically parameters for α-cells, as suggested by the need to scale α-cell signal transduction parameters, $k_{gA}$ and $k_{I}$ , to achieve the glucagon trajectories observed previously (8).

The release of glucagon by α-cells was considered along with its paracrine action on β-cells. In this study, glucose impacts glucagon response in isolated α-cells (21), and our results exhibit a U-shaped curve of glucagon *vs*. glucose in islets, again aligned with *in vitro* findings (18, 20). Notably, this response to rising levels of glucose is documented in a batch setting, where insulin transiently accumulates and suppresses the release of the α-cell hormone. The inhibition of glucagon secretion by insulin seen at low glucose levels eventually becomes saturated as insulin and glucose levels continue to rise leading to a concomitant surge in glucagon release. Hence, our findings underline the importance of relating the determination of hormone release to the batch or perifusion conditions employed.

The ability of glucose to stimulate glucagon secretion in isolated α-cells contradicts previously mentioned work that suggests otherwise (16–19). The ability to recreate results from these studies, using a first-principles model, highlights the need for further elucidation of the mechanisms underlying the function of α-cells. If, as these works suggest, glucose eventually is established to inhibit glucagon secretion directly with paracrine contributions, our model can easily be adapted by modifying Equation 4, highlighting the versatility of our approach. Besides the mode of interrogation of islet cell secretion, the number of islets tested is also important for the performance of the cells given the altered paracrine interactions. Based on the model reported here, the U-shape of glucagon secretion emerges and fades as the quantity of islets increases (**Figure 3**). Manifestation of this dependence is also evident in perifusion experiments where the secretion of insulin per islet decreases with larger numbers of islets (48) (see also below on the role of δ-cells).

Our work also suggests that insulin secretion is primarily stimulated by glucose at high glucose concentrations, and glucagon has little effect. However, at lower glucose levels (< 15 mM), insulin secretion slightly increased with the stimulation of glucagon (**Figure 5D**). The marginal increase is somewhat contrary to what is observed *in vitro* (6, 7). This difference likely stems from the difficulty in quantifying $m_{GB}$ , as the upper limit for glucagon’s contribution to insulin secretion is unclear, even experimentally. For example, an increase from 100 nM to 300 nM glucagon continued to stimulate insulin secretion in a recent report (7), suggesting that maximum secretion may not have been reached.

Analysis of the results from Zhu et al. (8) using the framework developed here shows a difference in glucagon response with a step increase in glucose between mice and human islets. However, when a scenario entailing mouse cells was ‘transformed’ to a theoretically equivalent one for human cells, both trends were recreated. This suggests a conserved interaction between glucagon and insulin that ultimately determines their secretion, regardless of both the experimental conditions (number of islets, flow rate, vessel volume, etc.) and the species examined. When compared to glucose stimulation, the signal propagation due to insulin and glucagon is faster likely due to the physical juxtaposition of α-cells and β-cells within the islets. The modeling effort was based on studies using isolated islet cells in culture. Yet, we employed the model to replicate the glucagon/insulin response of a whole pancreas. This analysis only served as an approximation, but our model prediction agrees with the observed hyperglucagonemia experienced by T1D patients (49). This is consistent with the lack of insulin, which suppresses glucagon release, due to β-cell ablation in T1D (21).

The model’s capacity to scale and replicate aspects of the whole pancreas function makes it suitable for use with computational multi-organ simulation platforms. By including modules of other organs and functions (e.g., liver, insulin clearance), a glucose feedback loop can be established mimicking glucose homeostasis in the human body, and leading to the development of more physiologically accurate algorithms for predicting the dose of insulin needed to be supplied dynamically, e.g., via an insulin pump. The extension of the model in this manner could possibly help to explain the variety of glucagon secretion trajectories observed *in vivo* (50). The precision of such system models can be enhanced through coupling to lab-on-a-chip technologies combining, for instance, β-cells with small intestine cells (51). The model with its direct relationship to easily measured variables (i.e., glucose, glucagon, and insulin concentrations), can provide complementary insights to previous whole-body level models (29).

The work also opens avenues for research on the relative release dynamics of insulin and glucagon. For example, $h_{IA}$ was found to be lower than $h_{GB}$ suggesting that glucagon secretion is more sensitive to insulin than the other way around. This implies that the synchronized production of insulin and glucagon is driven primarily by signaling effects of insulin (and glucose), instead of a more complex feedback loop (25). If insulin consistently suppresses α-cells, then glucagon’s stimulation of β-cells may improve blood sugar control at very low glucose concentrations. Viewing the islets as a controller of the glycemic setpoint (52), stimulation of insulin secretion could limit overshooting of the native setpoint due to excess glucagon secretion.

Our framework is amenable to the inclusion of other islet cell types, especially δ-cells, further expanding the scope of future investigations. Insulin can stimulate δ-cells through GABA (52) to secrete somatostatin, which inhibits the secretion of both insulin and glucagon. To this end, insulin is suggested to drive the synchronous pulses of somatostatin and insulin release (25). Additionally, the inhibitory role of somatostatin may help explain the previously mentioned observation that insulin secretion decreased with the number of islets (48), in the same way insulin influenced glucagon secretion. The link between somatostatin and glucagon secretion may also underpin the U-form of glucagon response, as somatostatin secretion is stimulated at lower glucose values than insulin (18, 20). Additionally, it has been observed that somatostatin inhibits glucagon secretion under normal conditions (17, 18). The β- and δ-cells are also connected through gap junctions (28), adding to the potential role of δ-cells. Indeed, another model has considered the paracrine regulation of glucagon secretion considering α-, β-, and δ-cells with an emphasis on their electrical activities (27). Furthermore, the hyperglucagonemia predicted here is higher than actual values in T1D patients (47), so the inclusion of δ-cells could yield the corrective suppression of glucagon secretion in this scenario. Developing mathematical models of paracrine interactions as the one reported here will aid the clarification of the roles of pancreatic hormones and glucose, and further our knowledge of pancreatic islet biology. Overall, this work adds to our understanding of the complex crosstalk between α- and β-cells in pancreatic islets and may provide a quantitative perspective on the functional role of glucagon and insulin interactions and secretion in glucose homeostasis in normal and pathological conditions.

## Data availability statement

Publicly available datasets were analyzed in this study. The code used for analysis can be found here: https://github.com/aedanbrown/Paracrine-Glucose-and-Insulin-in-Glucagon-Secretion.

## Author contributions

AB and ET contributed to the study concepts, design, and data analysis. Numerical results were generated by AB with the technical help of ET. AB wrote the first draft of the manuscript. All authors contributed to the manuscript revision, read, and approved the submitted version.

## Appendix

### Derivations of Equations 3 and 4

Originally, glucose and glucagon signals were assumed to have an additive effect:

$$X_{B}=\text{f}\left(X_{gB}\right)+\text{f}\left(X_{G}\right)$$

Because changes in glucose ultimately drive changes in insulin secretion, it was assumed that $\text{f}\left(X_{gB}\right)=X_{gB}$ :

$$X_{B}=X_{gB}+\text{f}\left(X_{G}\right)$$

Then, $\text{f}\left(X_{G}\right)$ was defined. Glucagon does not increase insulin secretion in the absence of glucose and glucose acts as an on-off switch for glucagon-stimulated insulin secretion (2, 6). With these in mind, a Hill function was used to act as this switch:

$$X_{B}=X_{gB}+\text{m}\left(X_{G}\right)\frac{X_{gB}^{n_{gB}}}{X_{gB}^{n_{gB}}+h_{gB}^{n_{gB}}}$$

The maximum of this Hill coefficient was set to 1 fulfill the on-off requirement.

Then, $\text{m}\left(X_{G}\right)$ was defined given that the addition of glucagon in perifusion experiments increases insulin secretion and with a downward concave profile (6) suggesting that the effect of glucagon may becoming saturated. A Hill function captures this trend, so one was used for $\text{m}\left(X_{G}\right)$ :

$$X_{B}=X_{gB}+\left(\frac{m_{GB}X_{G}^{n_{GB}}}{X_{G}^{n_{GB}}+h_{GB}^{n_{GB}}}\right)\left(\frac{X_{gB}^{n_{gB}}}{X_{gB}^{n_{gB}}+h_{gB}^{n_{gB}}}\right)$$

Finally, a basal signal intensity, $X_{B0}$ , was added as batch experiments reportedly have some amount of insulin secreted, even when no glucose is present (18). In the above equation, if $X_{gB}=0$ , then $X_{B}=0$ , which means that $R_{I}=0$ by Equation 1. Hence, the above equation becomes (Equation 3 in the text):

$$X_{B}=X_{gB}+\left(\frac{m_{GB}X_{G}^{n_{GB}}}{X_{G}^{n_{GB}}+h_{GB}^{n_{GB}}}\right)\left(\frac{X_{gB}^{n_{gB}}}{X_{gB}^{n_{gB}}+h_{gB}^{n_{gB}}}\right)+X_{B0}$$

Equation 4 was developed in a similar manner to Equation 3. As before, it was assumed that glucose and insulin independently contribute to the net signal $X_{A}$ :

$$X_{A}=\text{f}\left(X_{gA}\right)-\text{f}\left(X_{I}\right)$$

Again, it was assumed that the net signal is proportional to the glucose signal. Additionally, because glucagon acts in a saturating manner on $X_{B}$ , it was conjectured that insulin does as well on $X_{A}$ :

$$X_{A}=X_{gA}-\frac{m_{g}X_{gA}X_{I}^{n_{IA}}}{X_{I}^{n_{IA}}+h_{IA}^{n_{IA}}}$$

It is unclear if insulin can fully remove the contribution of glucose to the net signal, so we included the modulating term $m_{g}$ ; only $m_{g}\times 100\%$ of the glucose signal can be removed. To elaborate, if $m_{g}=0$ , then insulin has no impact on $X_{gA}$ . If $m_{g}=1$ , then insulin can fully reduce $X_{A}$ to zero if high enough insulin levels are present. Given that glucagon secretion eventually rises as it tracks a U-shape profile, it seems likely that insulin cannot fully remove the glucose signal, prompting the inclusion of $m_{g}$ as a term. Finally, a basal term $X_{A0}$ was added, and it was assumed that insulin could completely remove its effect:

$$X_{A}=X_{gA}-\frac{\left(m_{g}X_{gA}+X_{A0}\right)X_{I}^{n_{IA}}}{X_{I}^{n_{IA}}+h_{IA}^{n_{IA}}}+{\text{X}}_{\text{A}0}$$

Importantly, this basal term must be included in the equation for $X_{A}$ ; it cannot be included in the equation for $R_{G}$ . The contribution of $X_{A0}$ gives this expression the ability to decrease glucagon secretion at low glucose levels, even though $R_{G}$ increases with $X_{A}$ . It was assumed that this background signal could be completely abolished by insulin. At low glucose levels, $X_{gA}\approx 0$ , so $X_{A}$ can be simplified:

$$X_{A}=-\frac{X_{A0}X_{I}^{n_{IA}}}{X_{I}^{n_{IA}}+h_{IA}^{n_{IA}}}+X_{A0}$$

Because $X_{A0}$ was included, when the insulin levels increase as glucose increases, $X_{A}$ decreases. If the $X_{A0}$ term was not included, then $X_{A}\approx 0$ .

### Derivation of Equation 11

Initially, mass action kinetics were used to describe the transduction network depicted in **Supplemental Figure 6** (2). The following equations were derived:

$$\frac{dg_{i}}{dt}=c_{1}\text{g}-c_{2}\text{AD}P_{i}g_{i}-c_{0}g_{i}$$

$$\frac{dADP_{i}}{dt}=-c_{2}\text{AD}P_{i}g_{i}+c_{3}\text{AT}P_{i}-c_{8}\text{AD}P_{i}$$

$$\frac{dATP_{i}}{dt}=c_{2}\text{AD}P_{i}g_{i}-c_{3}\text{AT}P_{i}+c_{8}\text{AD}P_{i}$$

$$\frac{dK_{i}^{+}}{dt}=c_{4}K^{+}-c_{5}\left(ATP_{i}/ADP_{i}\right)K_{i}^{+}$$

$$\frac{dCa_{i}^{2+}}{dt}=c_{6}\left(K_{i}^{+}\right)\text{C}a^{2+}-c_{7}\text{C}a_{i}^{2+}$$

$\text{C}a^{2+}$ and $K^{+}$ were assumed to be approximately constant as they are in the bulk solution. This implies that the terms involving them can be simplified:

$$\frac{dK_{i}^{+}}{dt}=c_{4}-c_{5}\left(ATP_{i}/ADP_{i}\right)K_{i}^{+}$$

$$\frac{dCa_{i}^{2+}}{dt}=c_{6}\left(K_{i}^{+}\right)-c_{7}\text{C}a_{i}^{2+}$$

Here, $c_{6}$ and $c_{4}$ include the constant $K^{+}$ or $Ca^{2+}$ concentration, respectively. Importantly, $c_{5}$ should decrease with $ATP_{i}/ADP_{i}$ , and $c_{6}$ should increase with $K_{i}^{+}$ . Next, the transfer of $\text{C}a^{2+}$ was assumed to be the rate-limiting step, and all other steps achieve a pseudo-steady state. Using this assumption, an expression for $\text{AT}P_{i}$ can be obtained:

$$\text{AT}P_{i}=\frac{c_{2}ADP_{i}g_{i}+c_{8}ADP_{i}}{c_{3}}$$

Rearranging gives an expression for the ratio of $\text{AT}P_{i}$ to $\text{AD}P_{i}$ :

$$\frac{ATP_{i}}{ADP_{i}}=\frac{c_{8}}{c_{3}}+\frac{c_{2}}{c_{3}}g_{i}$$

In the cell, there is a large excess of ATP compared to ADP (53). Glucose metabolism is the primary source for this surplus of ATP (53). As a result, it was assumed that $\frac{c_{2}}{c_{3}}g_{i}\gg \frac{c_{8}}{c_{3}}$ , which suggests:

$$\frac{ATP_{i}}{ADP_{i}}\approx \frac{c_{2}}{c_{3}}g_{i}$$

Because $K^{+}$ transfer is equilibrated, an expression for $K^{+}$ can be obtained:

$$K_{i}^{+}=\frac{c_{4}}{c_{5}\left(ATP_{i}/ADP_{i}\right)}$$

Based on the expression for $\text{AT}P_{i}/\text{AD}P_{i}$ one can write:

$$K_{i}^{+}=\frac{c_{4}}{c_{5}\left(\frac{c_{3}}{c_{2}}g_{i}\right)}$$

Essentially, $K_{i}^{+}$ is a function of the interior glucose level, $g_{i}$ . Because $\text{C}a^{2+}$ transfer is not equilibrated, this expression for $K_{i}^{+}$ can be used in the equation for $\text{C}a^{2+}$ :

$$\frac{dCa_{i}^{2+}}{dt}=c_{6}\left(K_{i}^{+}\right)-c_{7}\left(Ca_{i}^{2+}\right)=c_{6}\left(\frac{c_{4}}{c_{5}\left(\frac{c_{2}}{c_{3}}g_{i}\right)}\right)-c_{7}Ca^{2+}$$

Here, $c_{6}$ increases with *K_i_^+^*, which in turn increases with $g_{i}$ . Therefore, we replaced $c_{6}\left(\frac{c_{4}}{c_{5}\left(\frac{c_{2}}{c_{3}}g_{i}\right)}\right)$ with $f\left(g_{i}\right)$ , a generic function representing an increase of the intracellular glucose. Assuming that glucose transfer via the glucose transporters is rapid, then $g_{i}\approx g$ , yielding:

$$\frac{dCa_{i}^{2+}}{dt}=\text{f}\left(g\right)-c_{7}\text{C}a_{i}^{2+}$$

If $\text{C}a_{i}^{2+}$ is replaced with some generic signal *X* and $c_{7}$ with a generic rate constant k this gives:

$$\frac{dX}{dt}=\text{f}\left(g\right)-\text{kX}$$

The following simple function satisfies the criteria for f(g) :

$$\text{f}\left(g\right)=\text{k}\frac{g}{g_{ba}}$$

This suggest that:

$$\frac{dX}{dt}=\text{k}\left(\frac{g}{g_{ba}}-X\right)$$

This equation becomes Equation 11 in the text when *X* is replaced with $X_{gB}$ , *k* is replaced with $k_{gB}$ , and the notation for concentrations is changed:

$$\frac{dX_{gB}}{dt}=k_{gB}\left(\frac{\left[g\right]}{{\left[g\right]}_{ba}}-X_{gB}\right)$$

Despite the simplicity of this expression, it affords flexibility in accommodating other signaling modalities as suggested for glucose control of glucagon secretion (3), potentially combined into a “net” pathway.
