# Supplementary material for: Mathematical modeling clarifies the paracrine roles of insulin and glucagon on the glucose-stimulated hormonal secretion of pancreatic alpha- and beta-cells
Source: Front Endocrinol (Lausanne). 2023 Aug 14;14:1212749. doi: 10.3389/fendo.2023.1212749 (PMC10461634; doi:10.3389/fendo.2023.1212749)
Supplement: Supplementary file 1 [file DataSheet_1.pdf]

Supplementary Material

Mathematical modeling clarifies the paracrine roles of insulin and glucagon on the glucose-stimulated hormonal secretion of pancreatic alpha- and beta-cells

Aedan Brown, Emmanuel S. Tzanakakis

* **Correspondence:** Emmanuel S. Tzanakakis: Emmanuel.Tzanakakis@tufts.edu

1.1 Supplementary Figures

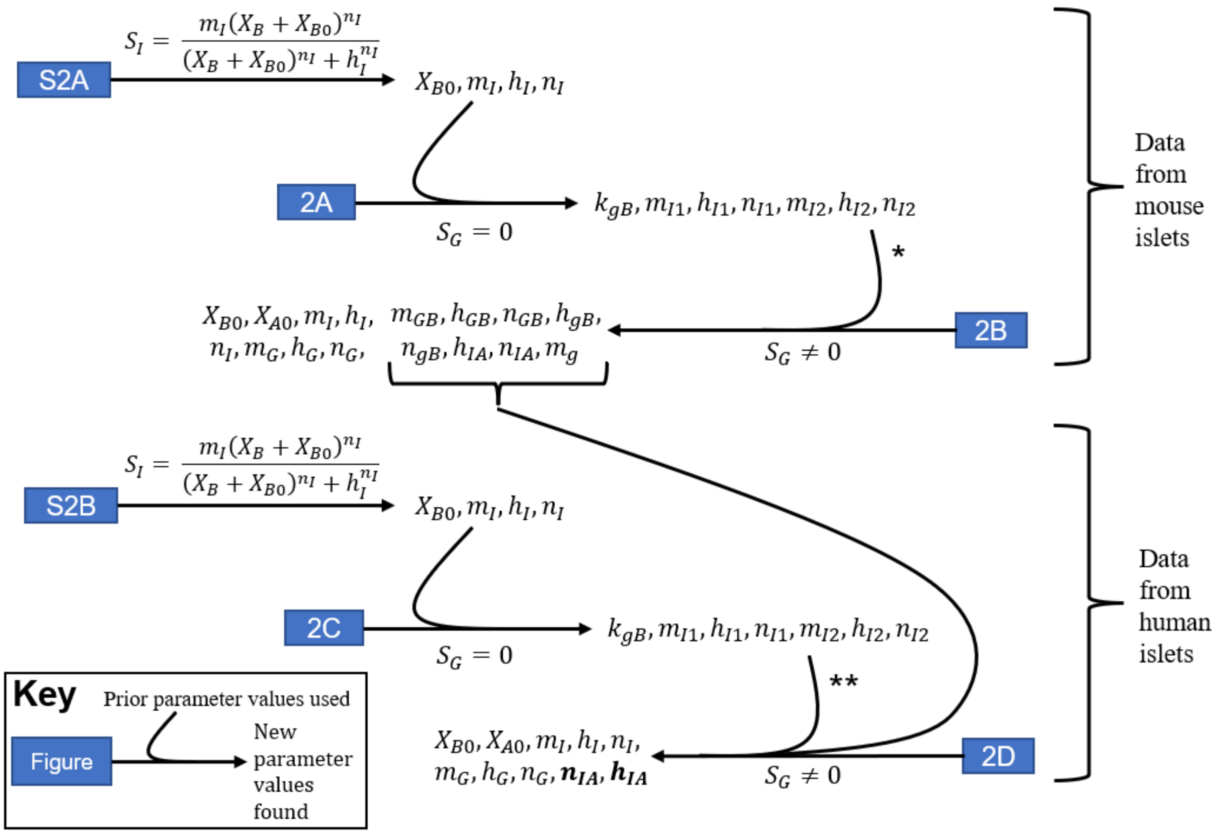

Supplemental Figure 1: Model parameter estimation workflow. As indicated by the key, blue boxes represent the figures with data which were used for computing parameter values. The incorporation of previously calculated/estimated parameters is also indicated. Initially, data from mouse islets were used, and the parameter values computed here were included in the calculations on human islet data. In the step using **Fig. 2D**, n_{IA} and h_{IA} are in bold, as they were allowed to vary. *: After the kinetic

parameters were calculated using **Figures 2A and 2C**, the rate constants of signal transduction were considered as equal ($k_{gB} = k_{gA} = k_G = k_I$), and the kinetic parameters were the same in β- and α-cells ($m_{I1} = m_{G1}$, $h_{I1} = h_{G1}$, etc.). **: Results from Zhu et al. (2019) were used for improved estimation of k_I and k_{gA}.

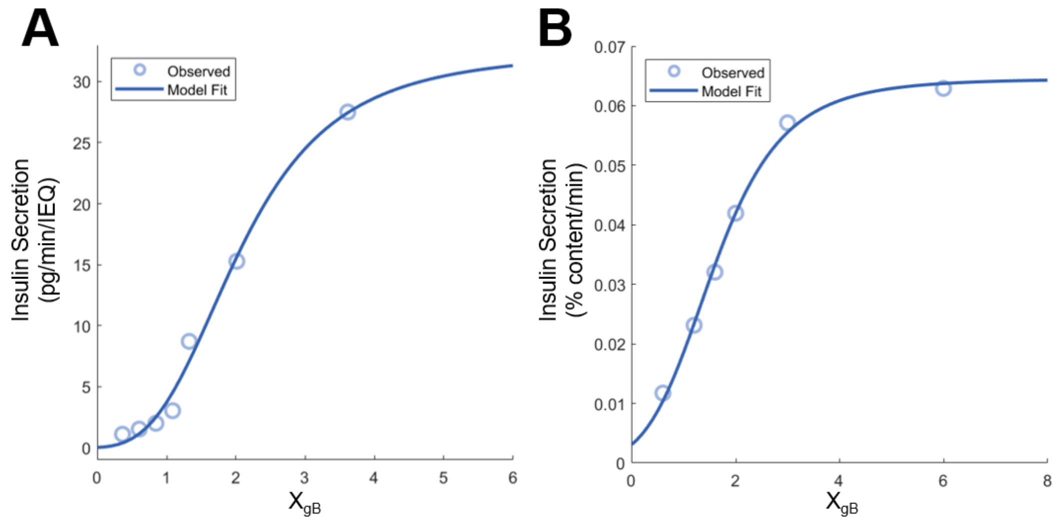

Supplemental Figure 2: The steady state insulin secretion values were used to determine model parameters in (A) mouse islets (Alcazar & Buchwald, 2019) and (B) human islets (Henquin et al., 2015).

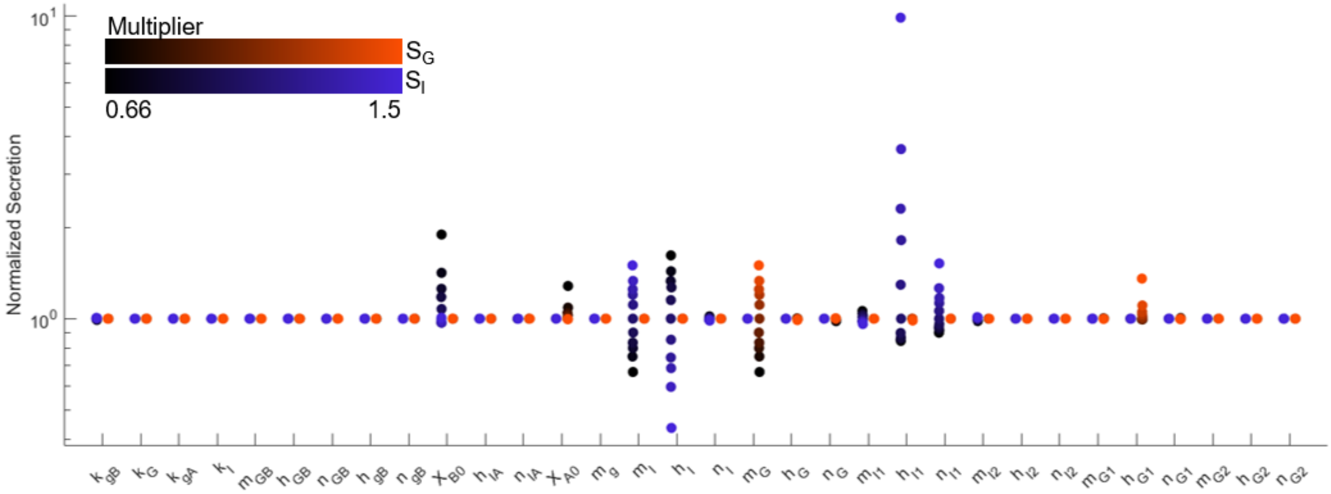

Supplemental Figure 3: Sensitivity analysis of the complete model in a perifusion setting. Each parameter was multiplied by a factor of 0.66 to 1.5, and the total insulin secretion (blue) and glucagon secretion (orange) was calculated in response to an increase from 1 mM to 15 mM of glucose in perifusion with 15 islets. Dark dots indicate factors closer to 0.66, while dots closer to blue or orange indicate a factor closer to 1.5.

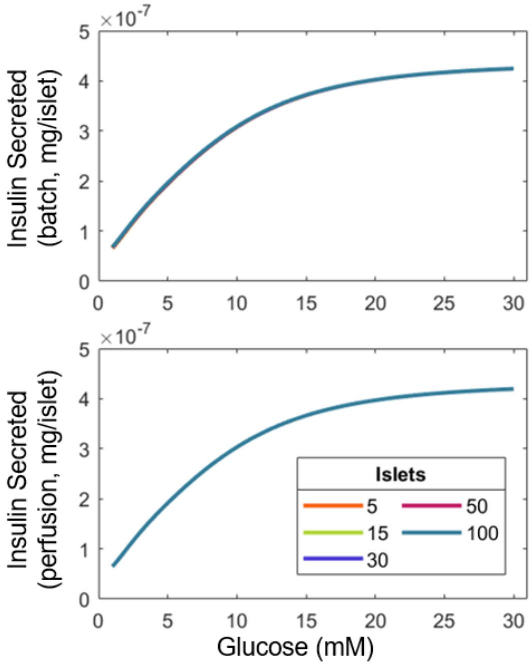

Supplemental Figure 4: Variation of islet number or static vs. dynamic interrogation of islets has little impact on the relationship between insulin and glucose release. Total insulin secretion in batch (top) and perifusion (bottom) modes with various numbers of islets in response to a step increase in glucose from 1 mM with 15 islets. Of note, there is significant overlap among the insulin response for different numbers of islets.

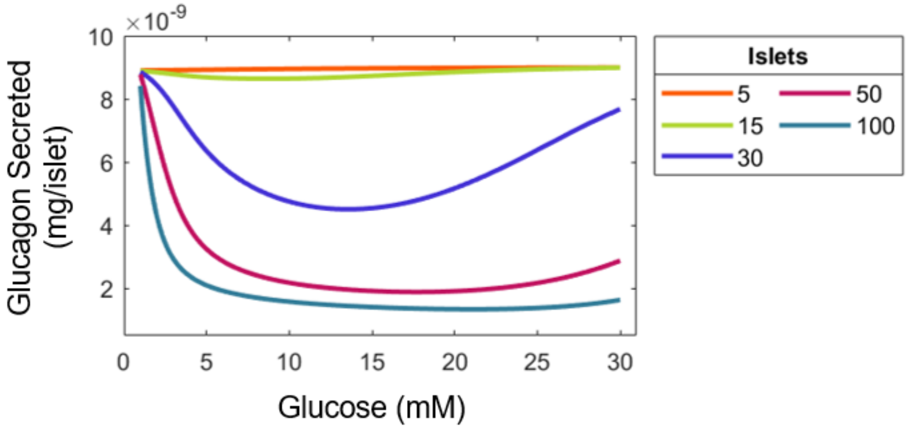

Supplemental Figure 5: At lower perifusion rates, the U-shape reappears. Glucagon secretion was measured per islet, as in **Figure 3B**, with a lower perifusion rate (0.0005 dL/min compared to 0.01 dL/min).

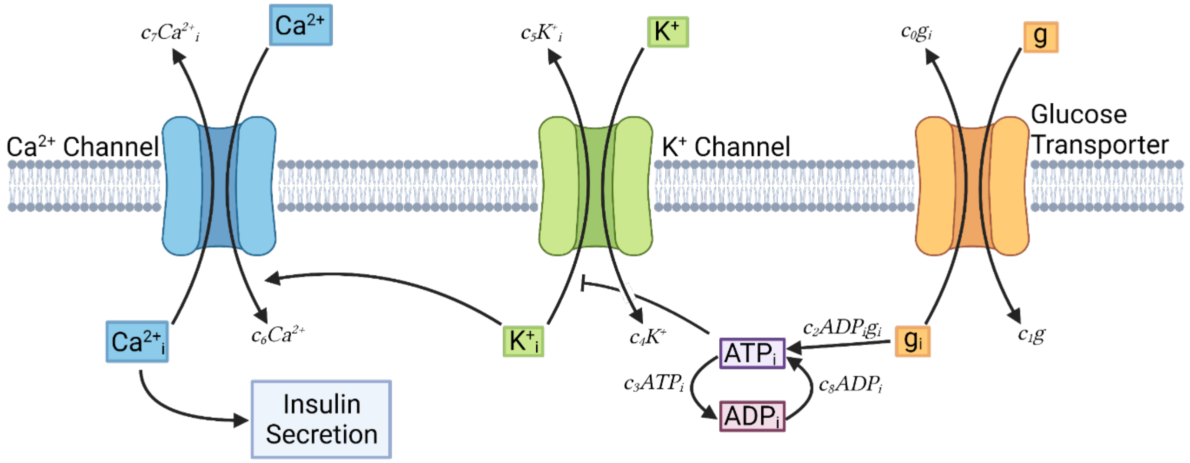

Supplemental Figure 6: Schematic of a simplified signal transduction network for insulin secretion triggered by glucose.

1.2 Supplementary Tables

Human Basal Glucose (mM)	5	Human Basal Glucagon (mg/dL)	2.5×10^{-6}	Human Basal Insulin (mg/dL)	2.2×10^{-5}
Glucose (mM)	16.7			Normalized Glucose	3.3
Glucagon Secretion (mg/min)	5×10^{-7}	Glucagon Concentration (mg/dL)	5×10^{-5}	Normalized Glucagon Concentration	20
Insulin Secretion (mg/min)	1.5×10^{-5}	Insulin Concentration (mg/dL)	1.5×10^{-3}	Normalized Insulin Concentration	68

Supplemental Table 1: An example of the analysis of the results in Zhu et al. (2019). The total glucagon and insulin secretion rates at steady state were order-of-magnitude estimated. The corresponding concentrations were calculated by using the mass balance for a generic species i at steady state: $[i] = S_i/\dot{Q}$, where S_i is the secretion rate of species i and $\dot{Q}$ is the perifusion rate. These values were then normalized by the basal levels in human islets. In this example, glucagon secretion rates slightly lowered as insulin secretion rates rose.
